# Inpainted Image Reconstruction Using an Extended Hopfield Neural Network Based Machine Learning System

**DOI:** 10.3390/s22030813

**Published:** 2022-01-21

**Authors:** Wieslaw Citko, Wieslaw Sienko

**Affiliations:** Department of Electrical Engineering, Gdynia Maritime University, Morska 81-87, 81-225 Gdynia, Poland; w.sienko@we.umg.edu.pl

**Keywords:** artificial intelligence, machine learning, image reconstruction and recognition

## Abstract

This paper considers the use of a machine learning system for the reconstruction and recognition of distorted or damaged patterns, in particular, images of faces partially covered with masks. The most up-to-date image reconstruction structures are based on constrained optimization algorithms and suitable regularizers. In contrast with the above-mentioned image processing methods, the machine learning system presented in this paper employs the superposition of system vectors setting up asymptotic centers of attraction. The structure of the system is implemented using Hopfield-type neural network-based biorthogonal transformations. The reconstruction property gives rise to a superposition processor and reversible computations. Moreover, this paper’s distorted image reconstruction sets up associative memories where images stored in memory are retrieved by distorted/inpainted key images.

## 1. Introduction

Machine learning, a sub-field of artificial intelligence, deals with algorithms that build mathematical models to automatically make decisions or predictions based on sample data called training sets. The concept of learning is the key to understanding intelligence in both biological brain structures and machines. The aim of machine learning is to create mappings $y=F\left(x\right)$, $y\in R^{m},x\in R^{n}$, generated by training sets $S={\left\{x_{i},y_{i}\right\}}_{i=1}^{N}$, the vectors of which are approximation nodes. Hence, the training points include:


(1) $$y_{i}=F\left(x_{i}\right),\;i=1\;,\;\ldots ,\;N$$

The machine learning model described in a previous paper [1] was derived from an extended Hopfield neural network and is based on spectral analysis that uses biorthogonal and orthogonal transformations. It should be emphasized that this system has a universal character that enables the implementation of basic functions of learning systems, such as pattern association, pattern recognition, and inverse modeling. One of the aforementioned properties of this model is the recognition and reconstruction of image patterns. In [1], we presented an example where the object of the reconstruction was an incomplete, inpainted image of a subject named Lena. Such examples of reconstruction allow for the development of a system based on the above-mentioned model of machine learning that can recognize people wearing masks. It is worth noting that the above-mentioned model of machine learning represents an alternative to classical image reconstruction/restoration systems, which make use of such processing tools as inverse modelling, deconvolution, Wiener filters, and PCA (Principal Component Analysis) [2,3,4,5].

Classical image reconstruction systems are currently being intensively supplemented and replaced by those using neural, neuro-fuzzy architecture, and algorithms, especially in medical applications [6,7,8,9,10]. A comprehensive review of recent advances in image reconstruction can be found in [11]. Current research is focused on sparsity, low-rankness, and machine learning [12,13]. It is worth noting that the most up-to-date image reconstruction structures are based on constrained optimization algorithms and adequate regularizers [14].

Recently, deep learning algorithms are driving a renaissance of interest in neural network research and applications (e.g., image processing). Most of the known deep learning algorithms are implemented in the form of ANN (DLNN) learned from training set data by minimizing loss functions. Thus, deep learning approach can be seen as a special topic in optimization theory. Standard types of deep learning neural networks include the multilayer perceptrons (MLP), convolutional neural networks (CNN), recurrent neural networks (RNN), and generative adversarial networks (GAN) [15,16,17,18]. However, optimal networks topology and implementation technology have not yet been selected (the generalizability of networks is not well understood, and there is a lack of explanation for the relationship between the network topology and performance [19]. Nevertheless, we claim that ANN should constitute both a universal algorithmic and physical models used in computational intelligence. It is clear that Hopfield-type neural networks are both physical and algorithmic models suitable for neural computations. Hence, we considered an extended Hopfield-type model of the neural network defined by the following equations:

(2) $$\dot{x}=\left(\eta W-w_{0}1+\varepsilon W_{s}\right)\theta \left(x\right)+I_{d}$$

where 

W—skew-symmetric orthogonal matrix;

$W_{s}$—real symmetric matrix;

1—identity matrix;

$\theta \left(x\right)$—vector of activation functions;

$I_{d}$—input vector; and

$\varepsilon ,\;w_{0},\;\eta$—parameters.

(3) $$\left(\eta W-{\mathrm{>w}}_{0}1+\varepsilon W_{s}\right)\theta \left(x\right)+I_{d}=0$$

An equilibrium equation of neural networks (2), i.e., gives rise to the universal models of machine learning based on biorthogonal transformations, enabling the realization of common learning systems functions. One of these functions is the implementation of associative memories. Thus, this paper’s inpainted image reconstruction system sets up associative memories where images stored in memory are retrieved by distorted/inpainted key images. To summarize, we propose a machine learning model that uses biorthogonal transformations based on spectral processing as alternative solutions to deep learning based on optimization procedures.

The rest of this paper is structured as follows: Section 2 provides details on the proposed learning algorithm and presents a structure of the machine learning systems for image processing. Section 2 contains also some results of computational verifications using MATLAB software. Section 3 includes some results of image processing as an inverse problem. Some unique properties of this machine learning system are discussed in Section 4. The conclusions underline the main features of the machine learning system presented in the article.

## 2. Materials and Methods

### 2.1. Machine Learning System for Image Processing

We consider a set of N black and white images represented by m rows and n columns, i.e., a set of $\left(m\cdot n\right)$ pixels with different shades of grayness. For vector analysis, each image is transformed by concatenating m rows to form the column vector $x_{i}\;\left(m\cdot n\times 1\right),\;i=1,\ldots ,\;N$. Thus, the set of N images is represented by the following matrix:

(4) $$X=\left[x_{1},x_{2},\;\ldots ,\;x_{N}\;\right],\dim x_{i}=m\cdot n=2^{k},\;k=3,\;4,\;\ldots ,\;$$

where

(5) $$N<\frac{1}{2}\left(n\cdot m\right).$$

The set of distorted images is given by the matrix:

(6) $$X^{\left(s\right)}=\left[x_{1}^{\left(s\right)},x_{2}^{\left(s\right)},\;\ldots ,\;x_{N}^{\left(s\right)}\;\right].$$

It is straightforward to observe that the training set is as follows:

(7) $$S={\left\{x_{i},x_{i}^{\left(s\right)}\right\}}_{i=1}^{N}.$$

S creates a mapping $F\left(\cdot \right)$ defined by the following properties:

(8) $$x_{i}=F\left(x_{i}\right)$$

and

(9) $$x_{i}^{\left(s\right)}\overset{F}{\to }x_{i},\;i=1,\;2,\;\ldots ,N.$$

Thus, the mapping F is implemented as a machine learning system for image reconstruction.

The structure implementing the mapping $F\left(\cdot \right)$ defined by Equations (8) and (9) can be obtained as the solutions of the equilibrium Equation (3). Thus, for $w_{0}=2,\;\varepsilon =1$ in Equation (3), one gets:

(10) $$\left(W_{2^{k}}-2\cdot 1+W_{s}\right)m_{i}+x_{i}^{\left(s\right)}=0$$

where $W_{2^{k}}^{2}=-1,$ skew-symmetric, orthogonal matrix

Hence, the N-solutions are as follows:

(11) $$m_{i}={\left(2\cdot 1-W_{s}-W_{2^{k}}\right)}^{-1}x_{i}^{\left(s\right)},\;i=1,\ldots ,\;N$$

where

(12) $$W_{s}=M{\left(M^{T}M\right)}^{-1}M^{T}$$

and

(13) $$M=\left\{m_{1},m_{2},\;\ldots ,m_{N}\right\}$$

is a spectrum matrix of given vectors $x_{i}$, i.e.,

(14) $$m_{i}=\frac{1}{2}\left(W_{2^{k}}+1\right)x_{i}$$

and

(15) $$x_{i}=\left(-W_{2^{k}}+1\right)m_{i},\;i=1,\ldots ,\;N.$$

Equation (11) can be seen as a determination of biothogonal transformation $T_{s}\left(\cdot \right):$

(16) $$m_{i}=T\left(x_{i}^{\left(s\right)}\right)$$

and Equation (14) can be seen as an orthogonal transformation:

(17) $$m_{i}=T\left(x_{i}\right),\;\;x_{i}=T^{-1}\left(m_{i}\right).$$

**Figure 1:**
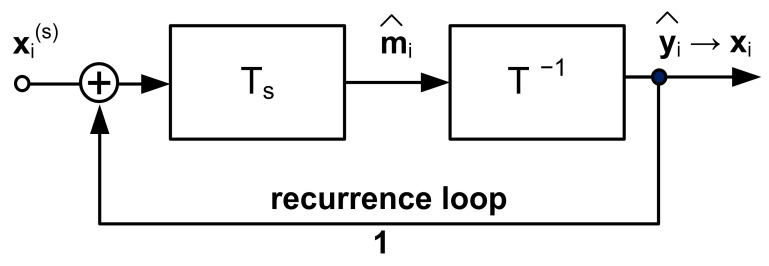
Structure of the machine learning model for image processing.

The transformations $T_{s}\left(\cdot \right)$ and $T^{-1}\left(\cdot \right)$, arranged as a realization of the mapping $F\left(\cdot \right)$, have the block structure, as shown in Figure 1 [1]. The orthogonal transformation $T\left(\cdot \right)$, which makes use of the Hurwitz-Radon matrix family [20], allows for determining the Haar–Fourier spectra of the system vectors $x_{i}$.

The structure from Figure 1 serves as the estimator of the spectrum $\left\{{\hat{m}}_{i}\right\}$:

(18) $${\hat{m}}_{i}=T_{s}\left(x_{i}^{\left(s\right)}\right),\;i=1,\;\ldots ,\;N.$$

In the system, due to the iterative nature of the feedback loop, the following convergence of vectors is obtained:

(19) $${\hat{m}}_{i}\to m_{i}$$

(20) $$\;{\hat{y}}_{i}\to x_{i},\;i=1,\;\ldots ,\;N.$$

The convergence determined by Equation (20) is performed in *K* iterations (*K* depends on the reconstruction problem, note the example shown below). Moreover, it should be noted that for input image $z\neq x_{i},\;i=1,\ldots ,\;N,$ the output of the system is given by the superposition of system vectors:

(21) $$F\left(z\right)=\sum_{i=1}^{N}\alpha_{i}x_{i},\;\alpha_{i}\in R.$$

The system vectors $x_{i}$ set up the attraction centers.

**Figure 2:**
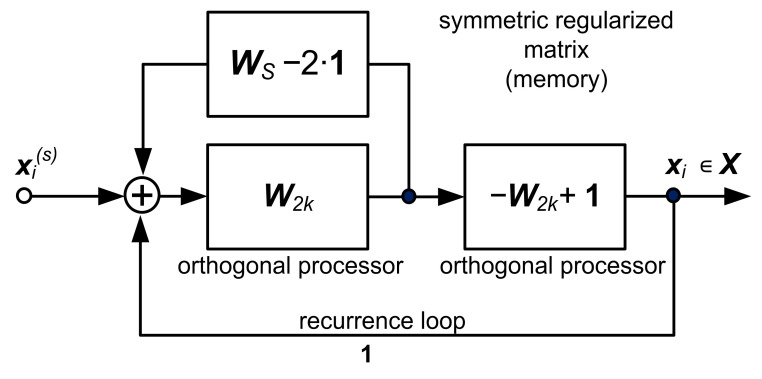
Block diagram of the approximator with lumped memory.

The structure in Figure 1 can also be represented as the lumped memory model in Figure 2. It is worth noting that this structure gives rise to the realization of an AI analog processor. However, this topic is beyond the scope of this paper. The synthesis algorithm of the system given in Figure 1 can be found in Appendix A

### 2.2. Computational Verification of the Learning Algorithm—Examples of Face Image Reconstruction and Person Recognition

**Figure 3:**
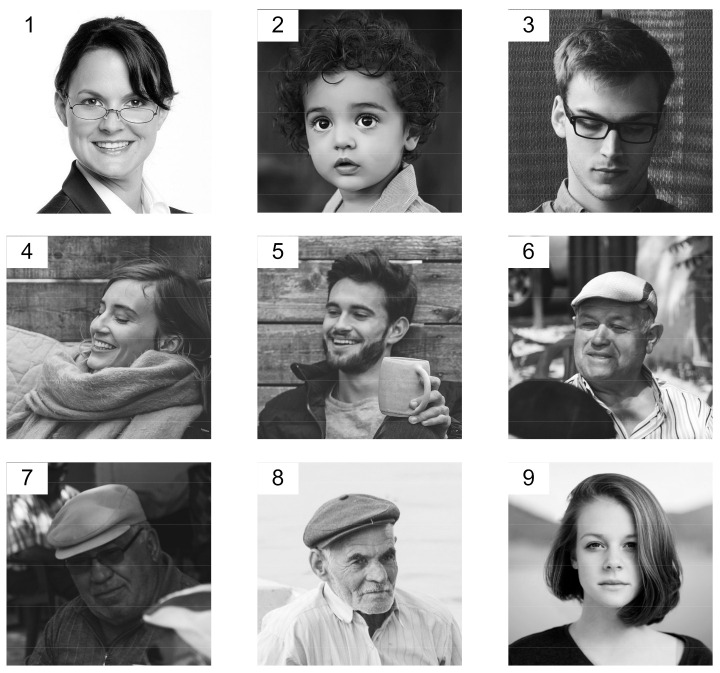
Face images saved (source: https://pixabay.com/pl, accessed on 17 February 2021).

A. The machine image processing system described in the previous section was used to reconstruct and classify a set of images. The system task was to reconstruct a complete face image based on a masked photo (mask applied by software) and to assign the reconstructed image to a specific person. In the system, photos of 9 faces $\left(N=9\right)$ were stored in memory in the form of a 64 × 64 matrix defining the degree of grayness of individual image pixels. The saved face images are presented in Figure 3. For vector analysis, each image was transformed by concatenating 64 lines into the form of a column vector $x_{i}\;\left(64\cdot 64\times 1\right),\;i=1,\ldots ,\;9$. After transformation, the set of 9 images was represented by the matrix $X\;\left(4096\times 9\right):X=\left[x_{1},x_{2},\;\ldots ,\;x_{9}\;\right],\dim x_{i}=64\cdot 64=4096=2^{k},\;k=12$.

**Figure 4:**
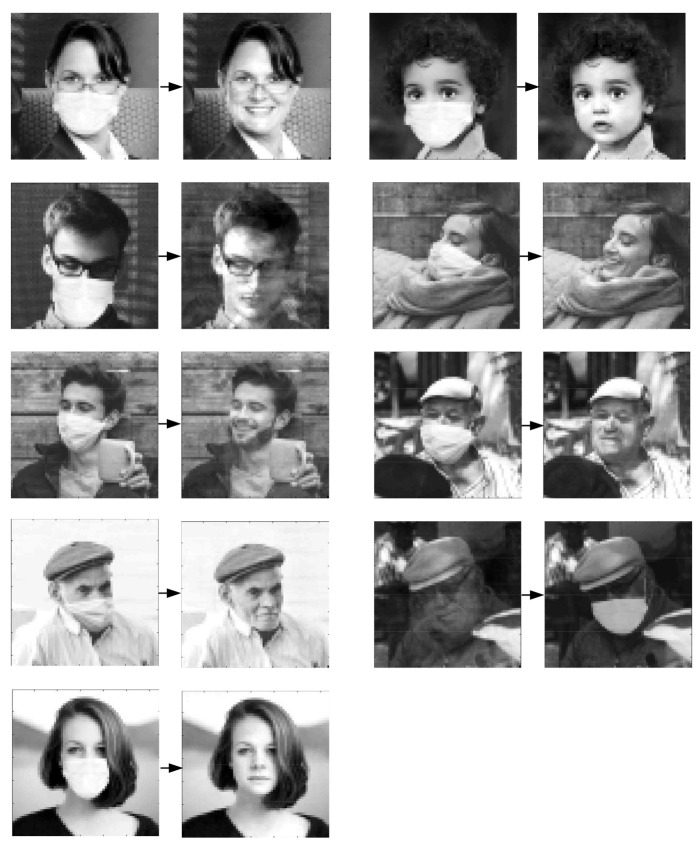
Reconstruction of face images of people wearing masks.

In the experiments, the identification numbers 1, 2, …, 9 were assigned to the images. The system vectors $u_{i}=\left[\begin{array}{c} x_{i}\\ i \end{array}\right],\;i=1,2,\;\ldots ,\;9$ were used to construct the machine learning system according to the procedure described in the previous section. Examples of the reconstruction of photos of people wearing masks are shown in Figure 4.

Table 1 shows the nominal values of the Recognition Index, i.e., the assigned numbers and their associated values after 100 iterations. The results presented in Table 1 show that in most cases, the value of the index rounded to the nearest integer corresponds to the nominal value. Thus, the system correctly identifies each person with the exception of Photo Number 3, where the person is incorrectly recognized. Increasing the number of iterations did not change the index, as the process quickly converges to the final value.

The convergence of the iterative process is illustrated in Table 2, which presents the index values obtained after successive iterations. The experiment was carried out for Photo Number 2 with the nominal value of the coefficient of 2.0.

**Figure 5:**
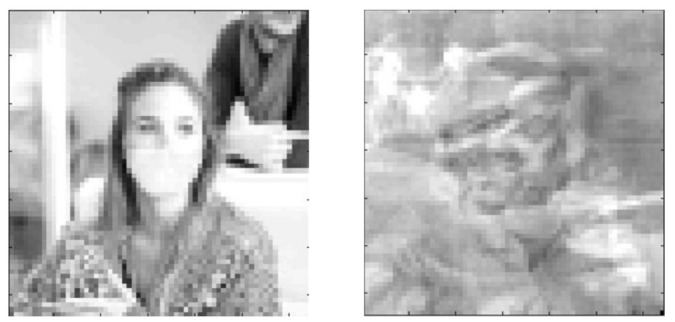
Attempt to recognize an unsaved photo.

A significant result that confirms the principle of the proposed system was obtained by substituting in a photo that was not saved in the system. The response shown in Figure 5 is a superposition of the photos stored in memory in the system (Equation (21)).

The mean squared error (MSE) values calculation for the previously performed image reconstructions are presented in Table 3. The second column of the table shows the MSE describing the difference between the original and masked photos, whereas the third column shows the error describing the difference between the original photo and the photo after reconstruction. Each time an image saved in the system was analyzed, the mean squared error decreased. For the reconstruction attempt shown in Figure 5, which uses an image not saved in the system, the MSE error is 2950.90.

The fractional value of the index in Table 1 reflects the system operation mechanism, which is a weighted combination of numbers 1, …, 9.

**Figure 6:**
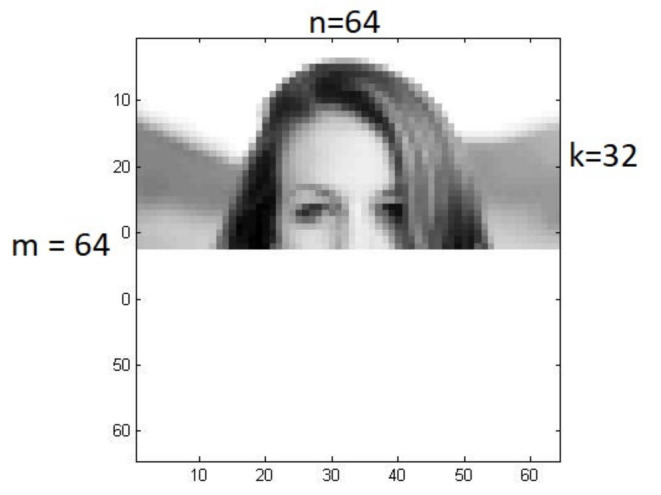
Masked image of the face in Photo Number 9 (Figure 3).

B. In the case of another masking method, as illustrated in Figure 6, a set of distorted images is given according to relationship (6) by the matrix:

(22) $$X^{\left(s\right)}=\left[x_{1}^{\left(s\right)},x_{2}^{\left(s\right)},\;\ldots ,\;x_{N}^{\left(s\right)}\;\right]$$

where $dimx_{i}^{\left(s\right)}=\left(k\cdot n\times 1\right),\;i=1,\ldots ,\;N,\;k<m$.

**Figure 7:**
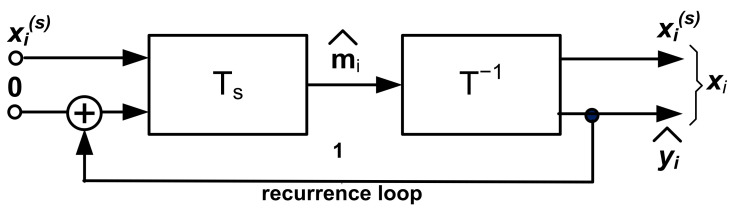
Structure of the reconstruction system when a fragment of the image (k–lines) is kept as the input.

The model structure for the reconstruction of such distorted images is shown in Figure 7.

**Figure 8:**
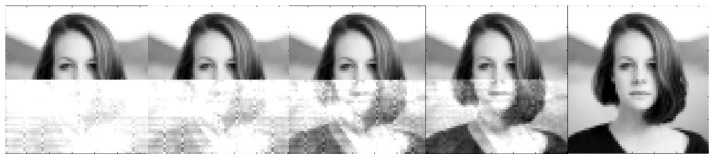
Image reconstruction process in Figure 6 (after 1, 2, 5, 10, and 100 iterations).

The image reconstruction process in Figure 6 is illustrated in Figure 8, which shows the results obtained after 1, 2, 5, 10, and 100 iterations. After 100 iterations, the reconstruction MSE is 0, and the identification index is 9.0. It is worth comparing the above values with the data for Photo Number 9 presented in Table 1 and Table 3.

**Figure 9:**
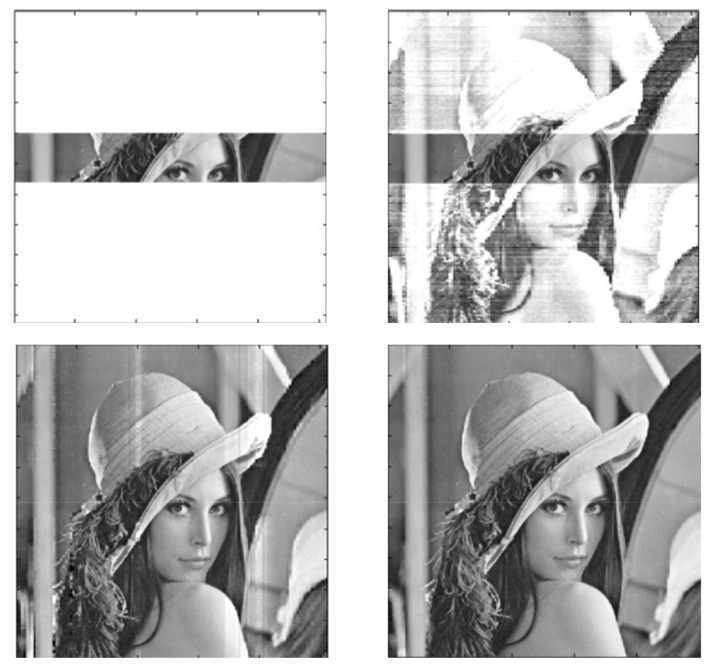
Image reconstruction of Lena’s photo (reconstruction system in Figure 7).

It is worth noting that, as mentioned in the Introduction, the reconstruction of Lena’ s photo was realized by using the structure presented in Figure 7 [1], as well. For example, one of the distorted images of Lena and the reconstruction is shown in Figure 9.

**Figure 10:**
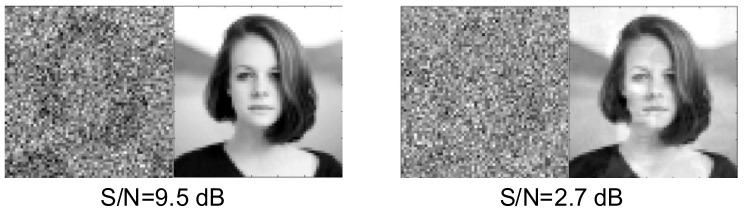
Reconstruction of distorted images (Items 10 and 14 in Table 4).

The potential reconstruction of a distorted image using the structure in Figure 1 is illustrated for Photo Number 9 (Figure 3) by superimposing a noise vector generated by using the RAND function in MATLAB. The measure of this distortion is the signal/noise ratio expressed in decibels. The results of such a reconstruction are presented in Table 4 and Figure 10.

Based on Table 4, the machine learning system correctly and automatically identified the distorted image at S/N > 10 dB. Yet, even at S/N = 2.7 dB in the reconstructed image, significant similarity to the saved original photo is observed.

**Figure 11:**
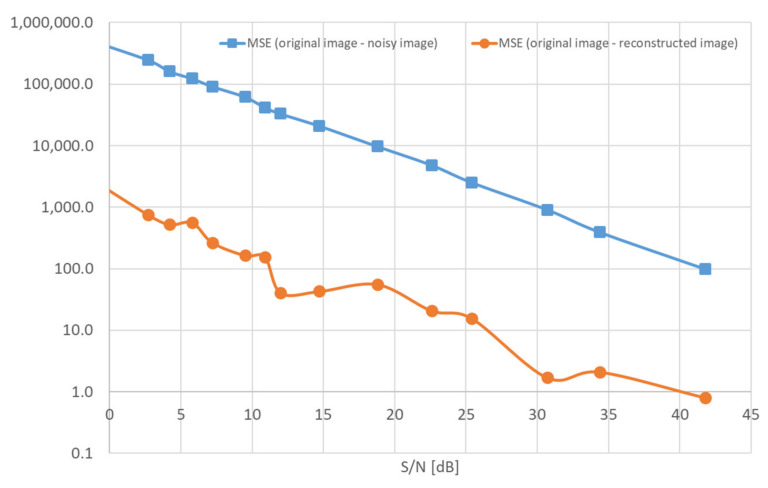
The plots of a function: MSE vs. S/N.

The numerical data in Table 4, set as a function, MSE vs. S/N, form the plots presented in Figure 11.

## 3. Inpainted Image Recognition and Reconstruction as an Inverse Problem

The image reconstruction models presented in the previous sections are based on the availability of training sets S in Equations (7) and (22) containing original and damaged patterns. Alternatively, a common model of image reconstruction is given by the equation:

(23) $$Ax=\tilde{y}$$

where

A—known processing operator, for example, A is a matrix;
x—original image; and
$\tilde{y}$—observed degenerate image.

According to Equation (23), the reconstruction of an image leads to solving the inverse problem. Most of the solutions to Equation (23) in the literature use an optimization solution [14,21], for example:

(24) $$\underset{x}{\min}||\tilde{y}-Ax{||}_{2}^{2},\;\;\;s.\;t.\;x\in K\;\;\mathrm{or}\;\underset{x}{\min}||\tilde{y}-Ax{||}_{2}^{2}+\beta R\left(x\right)\;\;$$

where

*K*—set of feasible solutions;
$R\left(x\right)$—regulizer;
β— regularization parameter.

As mentioned above, different types of neural networks are currently used to solve inverse problems in imaging, including image reconstruction. Many approaches to this problem can be found in recent reviews [22,23] and the novel proposal in [24].

The use of the machine learning model shown in Figure 1 to solve Equation (23) leads to the solution of the following problem:

(25) $$F\left(x\right):Ax=\tilde{y}\;\;x=F^{-1}\left(\tilde{y}\right)$$

where

$A-\left(m\times n\right)$ known real matrix, m≠n;
$\tilde{y}-\left(m\times 1\right)$ real matrix;
$x-\left(n\times 1\right)$ real matrix;
$m+n=2^{k},\;k=3,\;4,\;\ldots$

The case of m≠n is still under consideration. The generation of the training set $S={\left\{x_{i},y_{i}\right\}}_{i=1}^{N}$ for Equation (23) is given by:

(26) $$Ax_{i}=y_{i},\;\;i=1,\;2,\;\ldots ,N$$

where $x_{i},\;i=1,\;2,\ldots ,\;N$ is the vector form of training images, for example, those shown in Figure 3.

**Figure 12:**
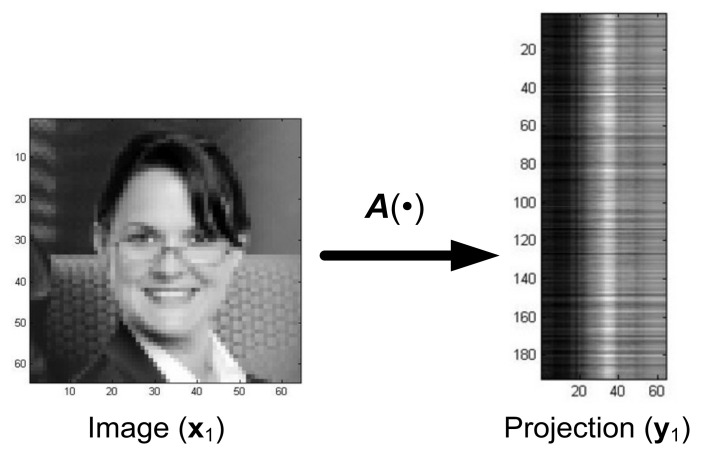
Original image and its transformation (projection).

Assuming that the matrix $A\;\left(m\times n\right)$ in projection (25) is a random matrix, the images $y_{i}$ of the training set become random vectors. For example, training image number 1 takes the form shown in Figure 12.

Thus, the vector form of the transformation of this image (No 1) is:

(27) $$Ax_{1}=y_{1}$$

where $A-\left(m\times n\right),m>n$.

**Figure 13:**
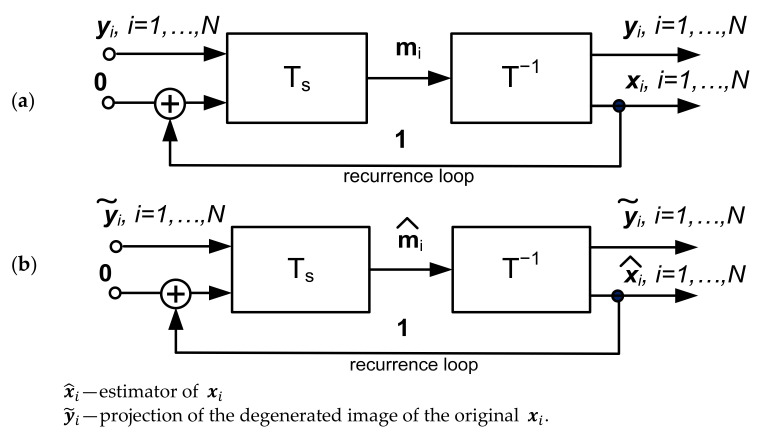
Structure of the system implementing inverse transformation. (**a**) $y_{i}$—undegenerated image projection; (**b**) ${\tilde{y}}_{i}$—degenerated image projection.

Taking the system vectors $u_{i}$ of the form

(28) $$u_{i}=\left[\begin{array}{c} y_{i}\\ x_{i} \end{array}\right],\;i=1,\;\ldots ,\;N,$$

the structure of the inverse mapping system (25), i.e.,:

(29) $$x_{i}=F^{-1}\left(y_{i}\right),\;i=1,\ldots \;,\;N$$

is given in Figure 13a,b.

It should be noted that the biorthogonal transformation $T_{s}\left(\cdot \right)$ and orthogonal transformation $T\left(\cdot \right)$ in Figure 13 are given by Equations (16) and (17), respectively:

Thus,

(30) $$m_{i}=T_{s}\left(\left[\begin{array}{c} y_{i}\\ 0 \end{array}\right]\right)$$

(31) $$u_{i}=T^{-1}\left(m_{i}\right)$$

where $u_{i}$—system vectors Equation (28).

**Figure 14:**
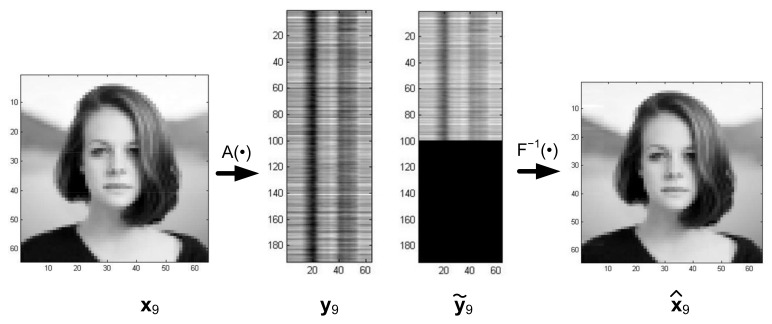
An exemplary reconstruction (F (·)—system from Figure 13b).

In the system presented in Figure 13, the distorted projections of the images ${\tilde{y}}_{i},\;i=1,\;\ldots ,\;N$ undergo reconstruction, in contrast with the system in Figure 7, where the distorted images are reconstructed. To illustrate the properties of the reconstruction system presented in Figure 13, a training set S was generated using Equation (26) consisting of nine images $x_{i},\;i=1,\;\ldots ,\;9,$ where $x_{i}$ were images from Figure 3, and their projections $y_{i},\;i=1,\;\ldots ,\;9$ were obtained with the random matrix A. An exemplary transformation of Image Number 9 from Figure 3 is shown in Figure 14.

In the system shown in Figure 13b, we obtain:

(32) $$||x_{9}-{\hat{x}}_{9}||{}_{2}^{2}=0.$$

To conclude, Figure 1, Figure 7, and Figure 11 show image reconstruction systems that substantially implement an associative memory structure for recognizing damaged key patterns. However, it is worth noting that on other hand, the system in Figure 13 implements inverse transformation and solves optimization tasks constrained by images stored in memory. Moreover, this system enables the solving of linear Equation (23) by using a random form of training vectors $x_{i}$ in Equation (26) [1] as well.

## 4. Discussion on Some Features of the Machine Learning System

**Figure 15:**
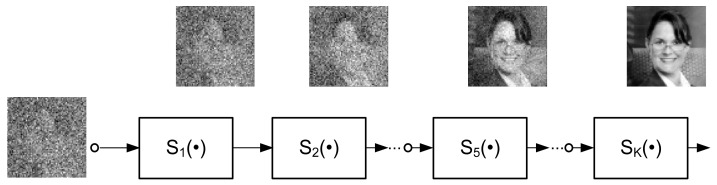
Multilayer learning structure (K—number of steps; e.g., K = 100).

A. This section focuses on some of the features that underlie the universality of the machine learning system presented in Figure 1 and Figure 7. First of all, it is clear that this machine learning system can be categorized as an iterative scheme. On other hand, the structure in Figure 1 can be treated as a feedforward block connection constituting a multilayer, deep learning architecture (Figure 15).

**Figure 16:**
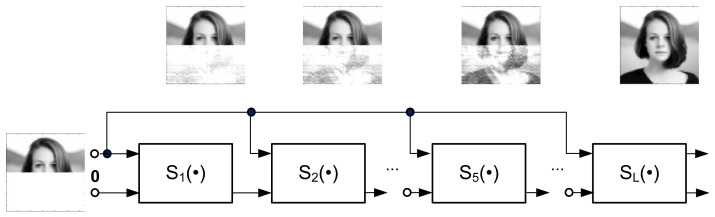
Multilayer learning structure (L—number of steps; e.g., L = 100).

The structure in Figure 7 can be similarly treated as a feedforward scheme, as shown in Figure 16.

The blocks $S_{i}\left(\cdot \right),\;i=1,\;\ldots ,\;K\left(L\right)$ are identical in multilayer structures (Figure 15 and Figure 16).

It is worth nothing that the multilayer structures in Figure 15 and Figure 16 can be seen as an implementation of deep learning using recurrent neural networks (RNN) [25]. However, the topology of these structures is not a result of optimization algorithms typically used to solve the inverse problems.

**Figure 17:**
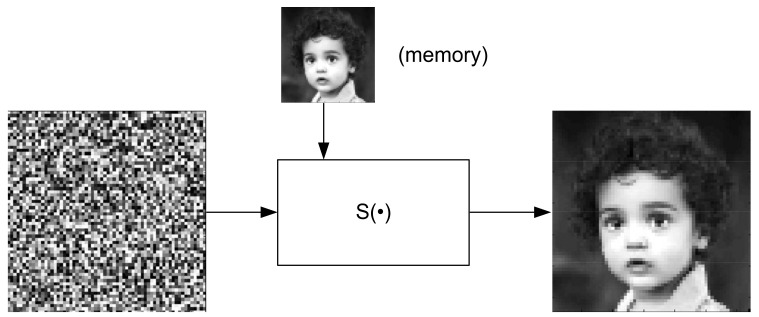
Illustration of global attractor properties.

B. An interesting property of the structure in Figure 1 can be set up by a computational experiment illustrated in Figure 17; i.e., when this structure memorizes only one image (e.g., photo No 2 in Figure 3), then any image is mapped on this memorized image (a property of global attractor).

C. Another interesting aspect of this machine learning system can be derived from the so-called Q-inspired neural networks feature [1]. This feature can be determined by the following statement:

Given a set of complex-valued training vectors ${\left\{x_{i},y_{i}\right\}}_{i=1}^{N}$ where $x_{i}\in C^{n},\;y_{i}\in C^{m},\;n+m=2^{k},\;k=3,\;4,\;\ldots$, a realization of mapping given by complex training vectors, i.e., $C^{n}\to C^{m}$ can be implemented as complex-valued neural networks or as a complex-valued machine learning system with the structure presented in Figure 2, where the memory block is determined by the Hermitian matrix $W_{H}\;(W_{s}\to W_{H}$ in Equation (3)).

**Figure 18:**
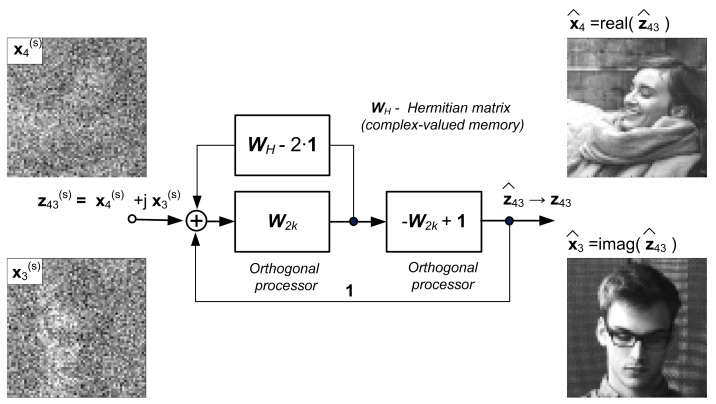
Complex-valued image reconstruction: $z_{43}=x_{4}+jx_{3\;},\;j^{2}=-1$, $x_{3},x_{4}$—vectorized form of images No. 3 and No. 4 in Figure 3, $x_{3}^{\left(s\right)},\;x_{4}^{\left(s\right)}$—distorted images.

Such a machine learning system can be used as an image processor to reconstruct complex-valued images. It is clear that the computational efficiency of this system is greater than that of the real-valued approximator (due to the processing of two images by only one system). Figure 18 provides an example of complex-valued image reconstruction.

D. This article focus on image processing by using the recursive machine learning system in Figure 1. It should be clear that this image processing sets up only one aspect of the potential system applicability in the field of signal processing. For example, the same machine learning could be used for time-series analysis and forecasting. To generalize, the essential function of the machine learning system described in this paper is the implementation of mapping defined by a training set $S={\left\{x_{i},y_{i}\right\}}_{i=1}^{N};i=1,\;\ldots ,N$, where $dimx_{i}=n,\;dimy_{i}=m.$ The recurrence is convergent under the linear independence of input vectors, and the number of vectors *N* fulfills (see Equation (5)) $N<0.5\;\left(n+m\right),\;n+m=2^{k},\;k=2,\;4,\;\ldots$. Thus, a large capacity system (large *N*) needs a large even dimension $\left(n+m\right)$ system. It could be considered as a disadvantage of this machine learning systems.

## 5. Conclusions

The aim of this article was to illustrate the potential for using the machine learning system shown in Figure 1 to reconstruct and recognize distorted or damaged patterns, in particular, images of people wearing masks. In contrast to the image reconstruction methods based on using optimization algorithms, this system employs the superposition of system vectors setting up asymptotic centers of attraction. Hence, this system is particularly useful for the implementation of associative memories. Thus, this paper’s inpainted image reconstruction sets up associative memories where images stored in memory are retrieved by distorted/inpainted key images. To conclude, we formulated another image processing tool augmenting the set of known image processing methods. Finally, all the image reconstructions presented in this paper, were done using MATLAB (The Math Works, Inc. MATLAB version 2021b).

## Figures and Tables

**Table 1:** Values of the Recognition Index of each person.

Photo Number	Index Nominal Value	Index Value after 100 Iterations
1	1.0	0.8622
2	2.0	1.6240
3	3.0	2.3660
4	4.0	3.9983
5	5.0	5.1259
6	6.0	5.8842
7	7.0	6.7262
8	8.0	8.0466
9	9.0	8.9576

**Table 2:** Convergence of the iterative process for Photo Number 2.

Number of Iterations	Index Value	Number of Iterations	Index Value
1	−0.0813	7	1.4607
2	0.1758	8	1.5394
3	0.5332	9	1.5843
4	0.8703	10	1.6078
5	1.1394	12	1.6233
6	1.3327	100	1.6240

**Table 3:** Values of the Recognition Index of each person.

Photo Number	MSE (Original Photo—Ask Photo)	MSE (Original Photo—Reconstructed Photo)
1	366.49	105.06
2	595.96	176.38
3	1573.00	570.95
4	398.00	37.58
5	552.04	114.55
6	675.67	112.13
7	828.53	221.09
8	171.52	26.05
9	327.06	40.75

**Table 4:** Mean squared error of reconstruction.

S/N Ratio [dB]	MSE (Original Image—Noisy Image)	MSE (Original Image—Reconstructed Image)	Index Value
41.8	98.6	0.8	9.04
34.4	391.9	2.1	8.97
30.7	909.3	1.7	9.01
25.4	2523.1	15.6	8.79
22.6	4807.1	20.7	8.78
18.8	9698.9	55.6	9.39
14.7	20,942.0	42.9	8.54
12.0	32,932.0	40.4	9.06
10.9	41,500.0	155.2	9.14
9.5	61,752.0	164.5	8.66
7.2	91,527.0	266.6	9.53
5.8	122,950.0	563.4	8.13
4.2	16,0360.0	521.0	7.14
2.7	24,6230.0	754.2	10.45
−2.5	62,9540.0	4375.8	11.17

## Data Availability

Not applicable.

## Appendix A

**Algorithm A1.** Summary of algorithm [1].
1 **Declaration:**
Input the set of training points:
$S=\;\left\{x_{i},\;y_{i}\right\},\;\;i=1,\;2,\;\ldots ,\;N$,
$x_{i}\in R^{n},\;y_{i}\in R^{m},\;n+m=2^{k},\;k=3,\;4,\;\ldots$
2. **System design**
Create system vectors $u_{i}:$
$u_{i}=\;\left[\begin{array}{c} x_{i}\\ y_{i} \end{array}\right]\;,\;dim\;u_{i}=n+m$.
Calculate the spectrum $m_{i}$ of system vectors $u_{i}:$
$m_{i}=\frac{1}{2}\left(W_{2^{k}}+1\right)u_{i}$
Create spectrum matrix M:
$M=\;\left[m_{1},\;m_{2},\;\ldots ,\;m_{N}\right]$
Calculate Hermitian matrix $W_{H}$:
$W_{H}=M{\left(M^{T}M\right)}^{-1}M^{T}$
Calculate orthogonal transformation $T\left(\cdot \right)$:
$T\left(\cdot \right)\;\equiv T=\frac{1}{2}\left(W_{2^{k}}+1\right)$
Calculate biorthogonal transformation $T_{s}\left(\cdot \right)$:
$T_{s}\left(\cdot \right)\;\equiv T_{s}={\left(2\cdot 1-W_{H}-W_{2^{k}}\right)}^{-1}$.
3. **Recursive procedure:**
*for i = 1:N*
${\tilde{x}}_{i}^{\left(0\right)}=0$
*while* $||{\tilde{x}}_{i}^{\left(l\right)}-{\tilde{x}}_{i}^{\left(l-1\right)}||\geq eps$
${\left[\begin{array}{c} {\tilde{x}}_{i}\\ y_{i} \end{array}\right]}^{\left(l\right)}=T^{-1}T_{s}\left(\left[\begin{array}{c} 0\\ y_{i} \end{array}\right]+{\left[\begin{array}{c} {\tilde{x}}_{i}\\ 0 \end{array}\right]}^{\left(l-1\right)}\right)$
*end*
*end*
(l=1, 2, … steps of recurrence)
$\mathrm{Final}\;\mathrm{results}\;\mathrm{of}\;\mathrm{recurrence}:\;{\tilde{x}}_{i}=x_{i}$.
